# Establishment of an Animal Model of Bisphosphonate-Related Osteonecrosis of the Jaws in Spontaneously Diabetic Torii Rats

**DOI:** 10.1371/journal.pone.0144355

**Published:** 2015-12-14

**Authors:** Kazuki Takaoka, Michiyo Yamamura, Toshihiro Nishioka, Tetsuya Abe, Joji Tamaoka, Emi Segawa, Masami Shinohara, Haruyasu Ueda, Hiromitsu Kishimoto, Masahiro Urade

**Affiliations:** 1 Department of Oral and Maxillofacial Surgery, Hyogo College of Medicine, Nishinomiya, Hyogo, Japan; 2 Planning and Development Section, CLEA Japan, Inc., Meguro-ku, Tokyo, Japan; 3 Laboratory of Immunobiology, School of Pharmacy, Hyogo University of Health Sciences, Kobe, Hyogo, Japan; INSERM - university Paris 7, FRANCE

## Abstract

**Background:**

We evaluated the side effects of bisphosphonate (BP) on tooth extraction socket healing in spontaneously diabetic Torii (SDT) rats, an established model of non-obese type 2 diabetes mellitus, to develop an animal model of BP-related osteonecrosis of the jaws (BRONJ).

**Materials and Methods:**

Male Sprague-Dawley (SD) rats and SDT rats were randomly assigned to the zoledronic acid (ZOL)-treated groups (SD/ZOL or SDT/ZOL) or to the control groups (SD/control or SDT/control). Rats in the SD/ZOL or SDT/ZOL groups received an intravenous bolus injection of ZOL (35 μg/kg) every 2 weeks. Each group consisted of 6 rats each. Twenty-one weeks after ZOL treatment began, the left maxillary molars were extracted. The rats were euthanized at 2, 4, or 8 weeks after tooth extraction, and the total maxillae were harvested for histological and histochemical studies.

**Results:**

In the oral cavity, bone exposure persisted at the tooth extraction site in all rats of the SDT/ZOL group until 8 weeks after tooth extraction. In contrast, there was no bone exposure in SD/control or SDT/control groups, and only 1 of 6 rats in the SD/ZOL group showed bone exposure. Histologically, necrotic bone areas with empty lacunae, microbial colonies, and less invasion by inflammatory cells were observed. The number of tartrate-resistant acid phosphatase-positive osteoclasts was lower in the SDT/ZOL group than in the SD/control group. The mineral apposition rate was significantly lower in the SDT/ZOL group compared with the SD/control group.

**Conclusions:**

This study demonstrated the development of BRONJ-like lesions in rats and suggested that low bone turnover with less inflammatory cell infiltration plays an important role in the development of BRONJ.

## Introduction

Bisphosphonates (BPs) are selectively taken up by osteoclasts and strongly inhibit bone resorption by inducing osteoclast apoptosis [1,2]. BPs are effective for the treatment of osteoporosis, Paget’s disease, multiple myeloma, hypercalcemia of malignancy, and osteolytic lesions of cancer metastasis [3–5]. Despite various benefits, however, the development of BP-related osteonecrosis of the jaws (BRONJ) has become an increasingly serious problem in a subset of patients [6,7]. BRONJ was first reported as a serious side effect of long-term BP treatment by Marx [8] in the United States in 2003. BRONJ is characterized by an area of uncovered bone in the maxillofacial region that does not heal for more than 8 weeks despite ordinary dental treatment in patients who are receiving or who have previously received BP therapy without prior craniofacial radiotherapy [9]. The risk factors for BRONJ can be classified as drug-related, local, and demographic/systemic. Drug-related risk factors include the potency of the specific BP. Zoledronic acid (ZOL) is more potent than pamidronate, and pamidronate is more potent than oral BPs. The intravenous (IV) route of administration results in greater drug exposure than the oral route. A longer duration of exposure appears to be associated with increased risk. Local and systemic risk factors for BRONJ include dentoalveolar surgery and tooth extractions, corticosteroid therapy, diabetes, smoking, alcohol use, poor oral hygiene, and chemotherapeutic drugs [10]. O’Ryan et al. [11] retrospectively reviewed healthcare databases, medical charts, and clinic files to identify all patients exposed to IV BPs who had a diagnosis of BRONJ. They reported that the most common clinical comorbidity in their cohort was diabetes mellitus (32.2%), which is considered a systemic risk factor for the development of BRONJ. Previous animal models of BRONJ associated with risk factors such as concomitant steroid use [12] and vitamin D deficiency [13] have been developed. To our knowledge, there have been no reports of experimental models of BRONJ associated with diabetes mellitus to date. In the present study, we focused on the risk factor of diabetes and studied the side effects of ZOL on tooth extraction socket healing and the development of BRONJ-like lesions in spontaneously diabetic Torii (SDT) rats, which is an established animal model of non-obese type 2 diabetes. Our main objective was to establish an animal model of BRONJ by testing the combined effects of diabetes and treatment with ZOL.

## Materials and Methods

### Ethics statement

All animal experiments were performed in compliance with the guidelines of the Animal Care and Use Committee of Hyogo College of Medicine in accordance with the Act on Welfare and Management of Animals (Law No. 105, Japan), the Standards Relating to the Care and Management of Laboratory Animals and Relief of Pain (Japanese Ministry of Environment, Notice No. 88, 2006), and the Fundamental Guidelines for Proper Conduct of Animal Experiment and Related Activities in Academic Research Institutions (Japanese Ministry of Education, Culture, Sports, Science and Technology, Notice No. 71, 2006). Our proposed study was approved by the committee under the institutional approval numbers of #B11-008.

### Animal handling

Thirty-six 5-week-old male SDT rats were provided for this study by CLEA Japan (Tokyo, Japan). Age-matched male Sprague-Dawley (SD) rats (CLEA Japan) were used as controls. The rats were housed in a light- and temperature-controlled environment. Food and water were available *ad libitum*. Body weight was measured once a week. Blood glucose levels were measured using a G-Checker (Gunze, Kyoto, Japan) every 2 weeks. Rats were anesthetized through inhalation of 2% isoflurane.

### Bisphosphonate

ZOL (2-[imidazol-l-yl]-1-hydroxyethylidene-1, 1-BP) was kindly provided by Novartis Pharma (Basel, Switzerland).

### Experimental methods and design

**Fig 1:**
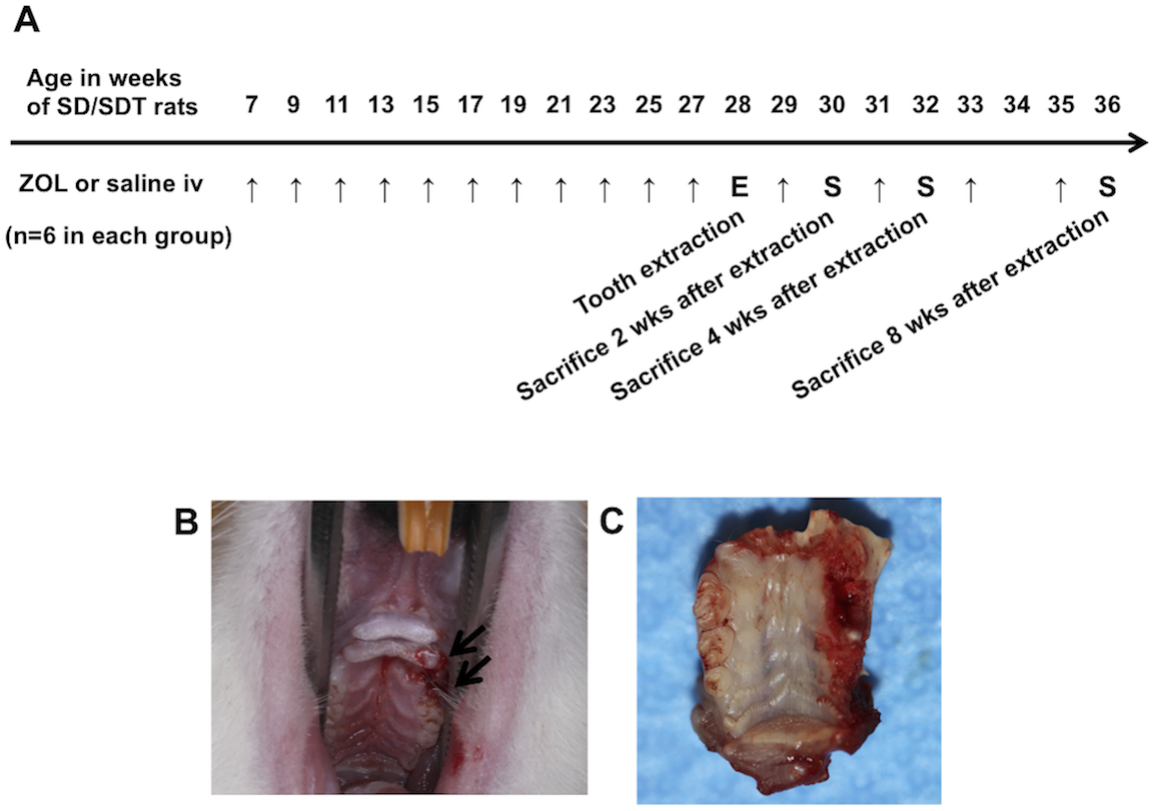
Schedule of treatment in SD and SDT rats. ZOL (35 μg/kg) was administered by an IV bolus injection via the tail vein every 2 weeks in the ZOL-treated groups, and the same volume of saline was administered in the control groups (**A**) (arrows = injections, E = tooth extraction, S = sacrifice). The left maxillary molars were extracted at 21 weeks after ZOL treatment was initiated (**B**, arrows). Then, at 2, 4, and 8 weeks after extraction, the total maxillae were harvested *en bloc* (**C**) and examined macroscopically and microscopically. Treatment with ZOL was continued until euthanasia.

After 2 weeks of acclimatization, 7-week-old SD and SDT rats were randomly divided into two groups. ZOL-treated groups (SD/ZOL or SDT/ZOL) rats received an IV bolus injection of ZOL (35 μg/kg) via the tail vein every 2 weeks (Fig 1A). The 35-μg/kg dose of ZOL used in this study was based on the dosage previously used by Hokugo et al. [13]. Control groups (SD/control or SDT/control) rats received saline solution in the same dosage volume as for ZOL treatment. Each group consisted of 6 rats each. The left maxillary molars were extracted at 21 weeks after ZOL treatment began (Fig 1B). Then, the total maxillae were harvested *en bloc* (Fig 1C) at 2, 4, and 8 weeks after tooth extraction. SD/ZOL and SDT/ZOL rats continued to receive treatment until euthanasia.

### Histological analysis

For euthanasia, the animals were anesthetized with pentobarbital (50 mg/kg; Dainippon Sumitomo Pharma Co., Osaka, Japan) and perfused with 4% paraformaldehyde in 0.1 M phosphate buffer through the left ventricle of the heart. The maxillae of the rats were removed, postfixed in the same fixative for 24 h, and decalcified in 10% ethylenediamine tetraacetic acid (EDTA) at room temperature for 2 weeks. Paraffin sections (4-μm thick) were cut using conventional methods and stained with hematoxylin and eosin (H&E). The numbers of empty osteocytic lacunae were counted within the alveolar bone 8 weeks after tooth extraction. Quantification was performed in five non-overlapping fields at a magnification of 400×.

### Assays for bone metabolism markers in serum

Blood samples were collected from the left ventricle for serum analysis 4 weeks after tooth extraction. The serum samples were used to measure C-terminal cross-linking telopeptide of type I collagen (CTX) and serum band 5 of tartrate-resistant acid phosphatase (TRACP-5b). CTX and TRACP-5b were determined by enzyme-linked immunosorbent assays (RatLaps ELISA, Nordic Bioscience Diagnostics, Herlev, Denmark; RatTRAP Assay, IDS, Inc., Fountain Hills, AZ, USA, respectively).

### Evaluation of bone phenotypes

To determine the bone morphometric parameters and microarchitectural properties of rat tibiae, tibia bones from 4 rats in each group were harvested at 4 weeks after tooth extraction, stored in 70% ethanol at 4°C, and then analyzed using a micro-CT scanner (Scan Xmate-L090; Comscan Techno Co., Ltd, Kanagawa, Japan). Scanning was conducted at 75 kV and 105 mA, with a spatial resolution of approximately 9.073 mm/pixel. For quantitative analysis, bone volume fraction (BV/TV, %), trabecular thickness (Tb.Th, μm), trabecular number (Tb.N, 1/mm), and trabecular separation (Tb.Sp, μm) were determined with the use of TRI/3D-BON software (RATOC System Engineering Co., Ltd., Tokyo, Japan).

### Dynamic calcein labeling and histomorphometry

Nine days and 3 days before sacrifice of the SDT rats, 4 rats in each group were given an intraperitoneal injection of calcein (10 mg/kg) for double labeling. Four weeks after tooth extraction, the maxilla of each rat was fixed in 70% ethanol without decalcification. Part of the maxilla samples were dehydrated and embedded in methyl-methacrylate (MMA; Wako Chemicals, Kanagawa, Japan). These plastic blocks were cut into 200-μm-thick sections using a precision bone saw. The sections were ground to a thickness of 15 μm using a precision lapping machine (Maruto, Tokyo, Japan), and calcein labeling was assessed. A Nikon microscope was used, and the pattern of fluorescence was analyzed with an inciting wavelength of 485 nm and an analyzing wavelength of 510 nm. The interlabel width was measured as the distance between the double fluorochrome labels. The mineral apposition rate (MAR, μm/day), defined as the distance between the midpoints of the double label divided by the number of days between calcein injections, was measured [14]. The remaining samples were dehydrated in ascending grades of ethanol and embedded in glycidyl methacrylate (GMA; Wako Chemicals). The plastic blocks were then cut into 3-μm-thick sections using a cutting machine (RM2255; Leica, Germany) for tartrate-resistant acid phosphatase (TRAP) staining. Samples were placed in 0.2 M acetate buffer (0.2 M sodium acetate and 50 mM L(+) tartaric acid in double-distilled H2O, pH 5.0) for 20 min at room temperature. Subsequently, the sections were incubated with 0.5 mg/ml naphthol AS-MX phosphate (Sigma-Aldrich Co., St. Louis, MO, USA) and 1.1 mg/ml fast red TR salt (Sigma) in 0.2 M acetate buffer for 1 to 4 h at 37°C until the osteoclasts appeared bright red [15]. The number of multinuclear TRAP-positive cells was counted in four non-overlapping fields of alveolar bone at a magnification of 200×.

### Statistical analysis

All data are expressed as the mean ± standard deviation (SD). The data were analyzed by one-way analysis of variance (ANOVA) followed by Dunnett’s test for determination of differences between groups. The non-parametric Mann-Whitney *U*-test was used to compare two groups. *P* values of <0.05 were considered to indicate statistical significance.

## Results

### Changes in body weight and nonfasting blood glucose levels

**Fig 2:**
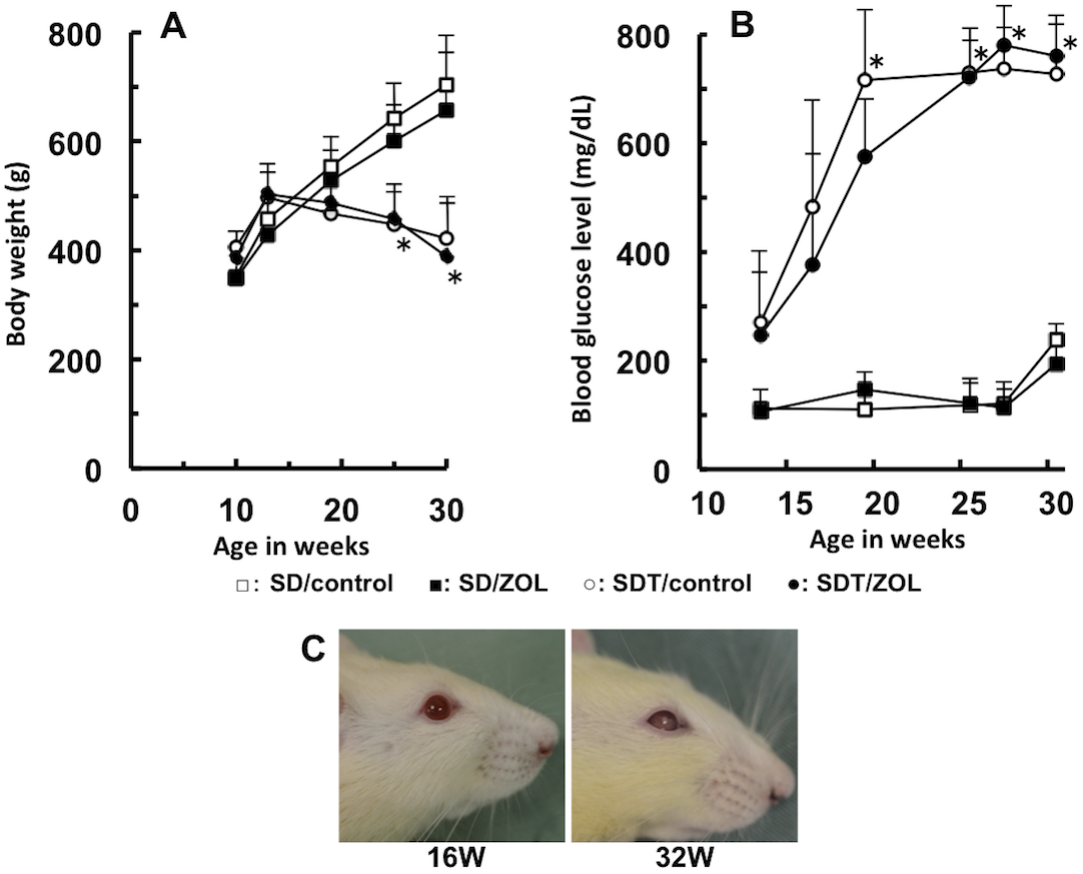
Validation of the diabetic phenotype of SDT rats. Changes in body weight (**A**) and blood glucose levels (**B**) in SD and SDT rats. □: SD/control, ■: SD/ZOL, ○: SDT/control, ●: SDT/ZOL. Data are expressed as means ± SD (n = 6). *p<0.005 versus SD/control. Macroscopic opacity of the lens observed at approximately 32 weeks of age in SDT rats (**C**).

Changes in the body weights of the SDT and SD rats are shown in Fig 2A. The mean body weight of SDT rats was similar to that of control SD rats until 14 weeks of age. Subsequently, the body weight of SDT rats gradually decreased with an increase in the incidence of diabetes. In contrast, the mean body weight of SD rats from 10 to 30 weeks of age increased throughout the experimental period. Changes in the body weights of SDT and SD rats in the ZOL-treated groups were similar to those in the vehicle-treated groups. Changes in the nonfasting blood glucose levels of SDT and SD rats are shown in Fig 2B. The nonfasting blood glucose level in SDT rats increased markedly and reached ≥600 mg/dL by 25 weeks of age. In SD rats, the nonfasting blood glucose level remained steady within the range of approximately 100–200 mg/dL. There was no significant difference in blood glucose levels between the ZOL-treated group and vehicle-treated group in either SD rats or SDT rats. Macroscopic opacity of the lens due to cataract, one of the ocular complications of diabetes [16], was observed at approximately 32 weeks of age in all SDT rats (Fig 2C).

### Macroscopic evaluation for the presence of BRONJ-like lesions

**Fig 3:**
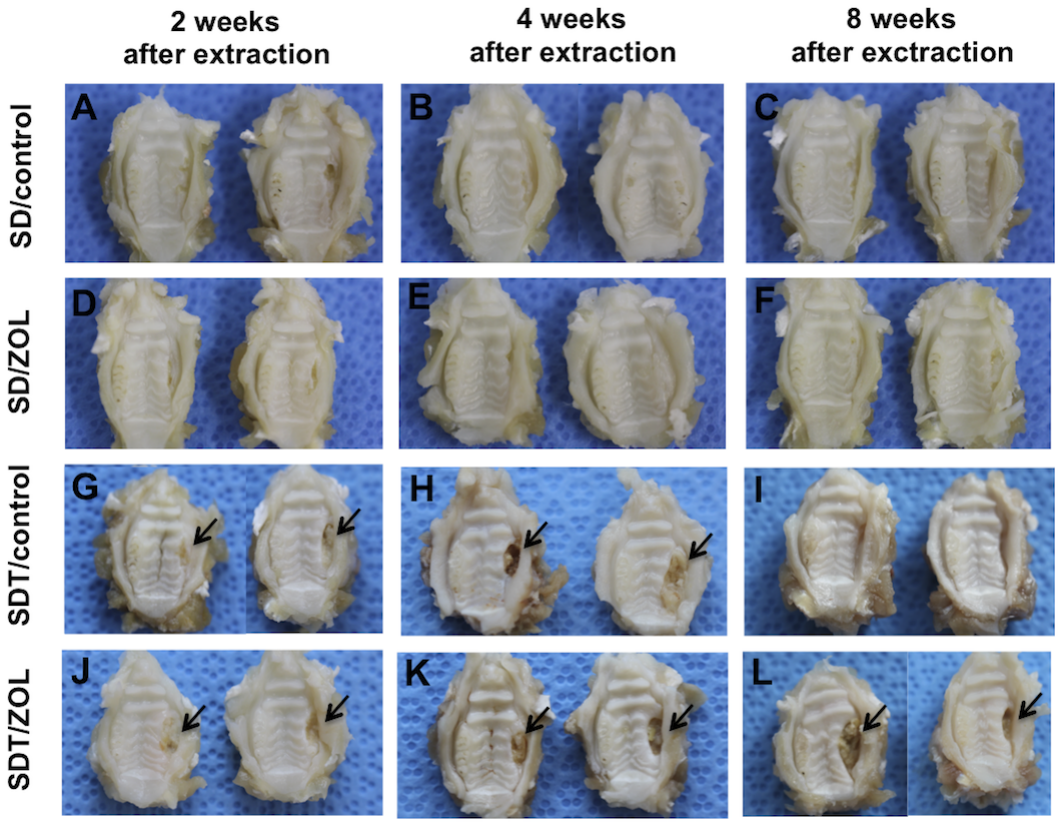
Macroscopic view of extraction sockets in SD/control (A-C), SD/ZOL (D-F), SDT/control (G-I), and SDT/ZOL (J-L) rats. Two representative specimens are shown. Normal healing after molar extraction was observed in SD/control (**A**) and SD/ZOL (**D**) rats by 2 weeks after extraction. Extraction sockets in both groups were covered with intact epithelium at 4 weeks (**B, E**) after extraction and at 8 weeks (**C, F**) after extraction. Apparent mucosal disruption with exposed bone (arrows) was seen at the extraction site at 2 (**G**) and 4 (**H**) weeks, but not at 8 weeks (**I**) after extraction in the SDT/control group and at 2 (**J**), 4 **(K**), and at 8 weeks (**L**) after extraction in the SDT/ZOL group.

Gross evidence of extraction socket healing was confirmed by 2 weeks in the majority of SD rats, regardless of ZOL treatment (Fig 3A–3F). In contrast, open wounds and bone exposure were noted in most SDT/control rats and all SDT/ZOL rats at 2 and 4 weeks after extraction (Fig 3G, 3H, 3J and 3K). Extraction sockets in SDT/control rats had completely healed by 8 weeks after extraction (Fig 3I). However, bone exposure was consistently found in all SDT/ZOL rats at 8 weeks (Fig 3L, Table 1).

**Table 1:** Incidence of bone exposure at 8 weeks after tooth extraction by ZOL treatment.

	Bone exposure		Empty osteocyte lacunae of alveolar bone per unit tissue area
	Macroscopic	Microscopic	
SD/control	0/6	0/6	6.8±1.8
SD/ZOL	0/6	1/6	17.8±4.9*
SDT/control	0/6	0/6	8.5±3.2
SDT/ZOL	6/6	6/6	36.6±7.8*

*p<0.05 versus SD/control.

### Histological evaluation of BRONJ-like lesions

**Fig 4:**
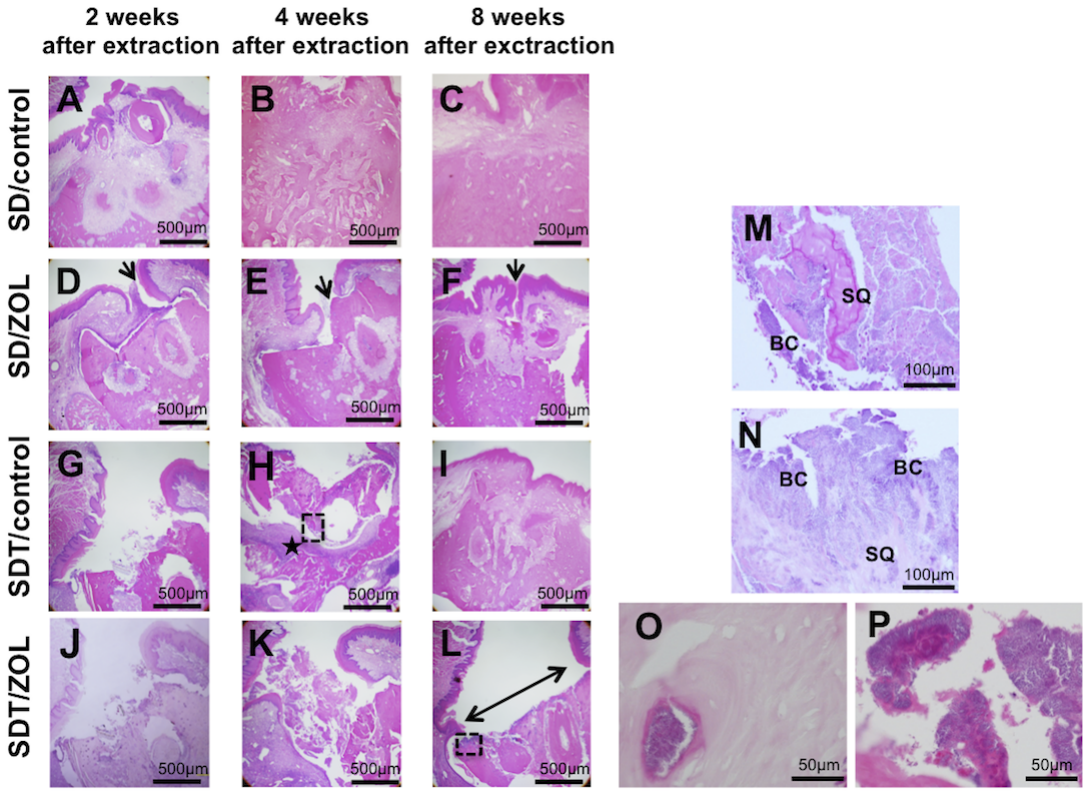
Photomicrographs of extraction sockets in SD/control (A-C), SD/ZOL (D-F), SDT/control (G-I), and SDT/ZOL (J-L) rats. Healed gingival mucosa with complete epithelial coverage in the SD/control group. (**D, E**) Partial deficiency of epithelial coverage at 2 and 4 weeks in the SD/ZOL group (arrow). (**F**) Nonkeratinized oral epithelium grew towards the sequestrum at 8 weeks after tooth extraction in 1 of 6 SD/ZOL rats (arrow). (**G**) Unhealed open socket with an area of exposed bone and no mucosal coverage at 2 weeks after extraction in the SDT/control group. (**H**) Interstitial tissue (★) under bone sequestra at 4 weeks after extraction. (**I**) Healed gingival mucosa with complete epithelial coverage at 8 weeks after extraction in the SDT/control group. (**J, K**) Unhealed open sockets with an area of exposed bone and no mucosal coverage at 2 and 4 weeks after extraction in the SDT/ZOL group. (**L**) Open sockets without epithelial lining (left right arrow) at 8 weeks after extraction in the SDT/ZOL group. H&E stain, original magnification, ×40. **Photomicrographs of magnifying dotted square area in (H) and (L)**. (**M**) Necrotic bone sequestra (SQ) with empty osteocyte lacunae covered with bacterial colonies (BC) and marked inflammation in (H) of SDT/control rat. (**N**) Necrotic bone sequestra (SQ) with empty osteocyte lacunae covered with bacterial colonies (BC) and less inflammation in (L) of SDT/ZOL rat. H&E stain, original magnification, ×200. **(O)** High magnification of empty osteocyte lacunae and **(P)** bacterial colonies in SDT/ZOL rats. H&E stain, original magnification, ×400.

Sections of the extraction sockets were stained with H&E and examined histologically at 2, 4, and 8 weeks after tooth extraction in all four groups (Fig 4). Complete epithelial coverage was noted in the SD/control group at 2 weeks of extraction, despite the presence of root fragments (Fig 4A). The tooth extraction socket was filled with new bone at 4 weeks (Fig 4B) and had healed normally at 8 weeks after extraction (Fig 4C). In contrast, 3 of 6 SD/ZOL rats showed unhealed gingival epithelium accompanied by delayed bone formation in the extraction socket, where necrotic bone was observed with mild inflammatory cell infiltration at 2 and 4 weeks after extraction (Fig 4D and 4E). The extraction socket healed with complete epithelial coverage in 5 of 6 SD/ZOL rats at 8 weeks after tooth extraction. In one SD/ZOL rat, although the edge of the wound in the oral mucosa appeared to close, non-keratinized oral epithelium grew towards the sequestrum at 8 weeks after tooth extraction (Fig 4F).

The SDT/control group and the SD/ZOL group both showed a delayed closure of the extraction socket at 2 weeks after tooth extraction (Fig 4G and 4J). In contrast to the SD/ZOL group, bone sequestration, marked inflammation, and bacterial colonies were observed in the SDT/control group at 4 weeks after extraction (Fig 4H and 4M). The extraction socket healed with complete epithelial coverage at 8 weeks after extraction (Fig 4I). In contrast, open sockets with areas of exposed bone were seen in the SDT/ZOL group, even at 8 weeks after extraction. Histologically, these areas showed a lack of epithelial lining in the alveolar socket. Areas of necrotic bone with empty lacunae, bacterial colonies, and mild inflammation were observed (Fig 4L and 4N–4P). The number of empty osteocyte lacunae in alveolar bone was significantly increased by ZOL treatment (Table 1).

### ZOL treatment on reduces bone metabolism markers in serum

CTX and TRACP-5b levels were decreased by ZOL treatment in SDT and SD rats. The TRACP-5b levels in the SD/ZOL, SDT/control and SDT/ZOL groups were significantly lower than in the SD/control group (Table 2).

**Table 2:** Changes in bone metabolizing markers in serum at 4 weeks after tooth extraction by ZOL treatment.

	CTX(ng/ml)	TRACP-5b(U/L)
SD/control	44.3±8.7	1.43±0.27
SD/ZOL	40.2±11.5	0.89±0.26*
SDT/control	38.8±12.1	1.00±0.17*
SDT/ZOL	35.5±9.8	0.74±0.26*

*p<0.05 versus SD/control.

### Bone histomorphometric analysis of the proximal tibia

Bone histomorphometric analysis was used to determine BV/TV, Tb.Th, Tb.N and Tb.Sp (Table 3). There were no differences in BV/TV, Tb.Th, Tb.N and Tb.Sp between SD rats and SDT rats. BV/TV and Tb.N were significantly increased by ZOL treatment in both SD rats and SDT rats. Tb.Th was increased, but not significantly by ZOL treatment. In addition, the Tb.Sp level was significantly decreased by ZOL treatment in both SD rats and SDT rats.

**Table 3:** Bone histomorphometric analysis of the proximal tibia

	Parameter			
	BV/TV(%)	Tb.Th(μm)	Tb.N(1/mm)	Tb.Sp(μm)
SD/control	24.97±5.15	75.20±13.11	3.31±0.79	247.20±98.11
SD/ZOL	58.92±8.85* ^,^ **	122.53±19.97	5.92±0.82* ^,^ **	150.53±23.97* ^,^ **
SDT/control	22.38±6.56	65.93±6.24	3.38±0.56	238.93±109.24
SDT/ZOL	40.14±10.85* ^,^ **	104.26±21.56	5.14±1.05* ^,^ **	134.21±51.13* ^,^ **

*p<0.05 versus SD/control.

**p<0.05 versus SDT/control.

### Dynamic parameters

**Fig 5:**
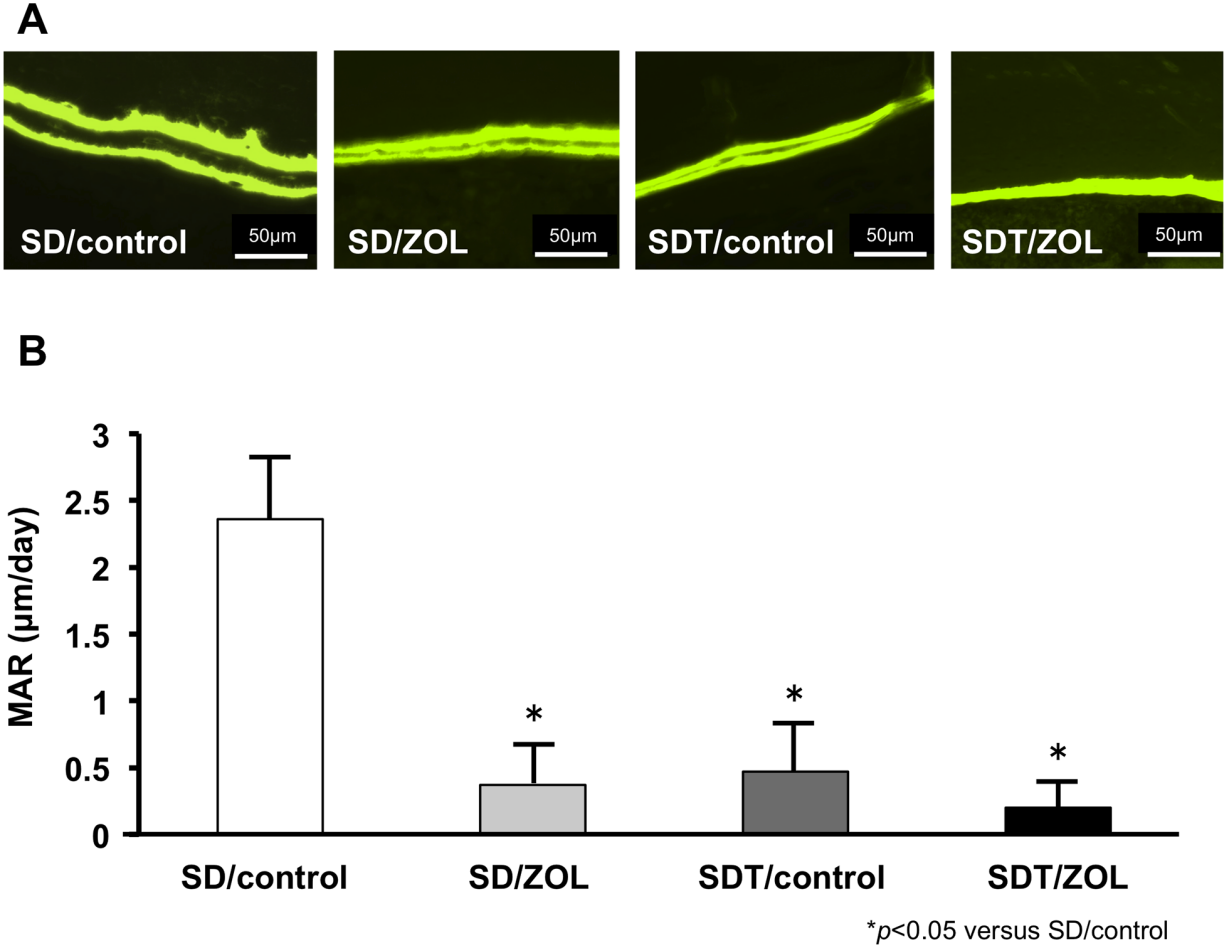
Fluorescence photomicrographs of calcein bone labeling at 4 weeks after tooth extraction. **(A)** Images of calcein double labeling of the upper alveolar bone. Original magnification, 400×. **(B)** Mineral apposition rate (MAR) measured at around the upper alveolar septum.

After calcein injection, two clear fluorescent lines were seen in the bone in the SD rats and SDT rats (Fig 5). Two clear calcein-labeled lines were recognizable in the newly formed bone around the upper alveolar bone. Analysis of the formation parameter revealed that the SD/control group presented a higher MAR compared with the SD/ZOL, SDT/control and SDT/ZOL group.

### Osteoclast activity

**Fig 6:**
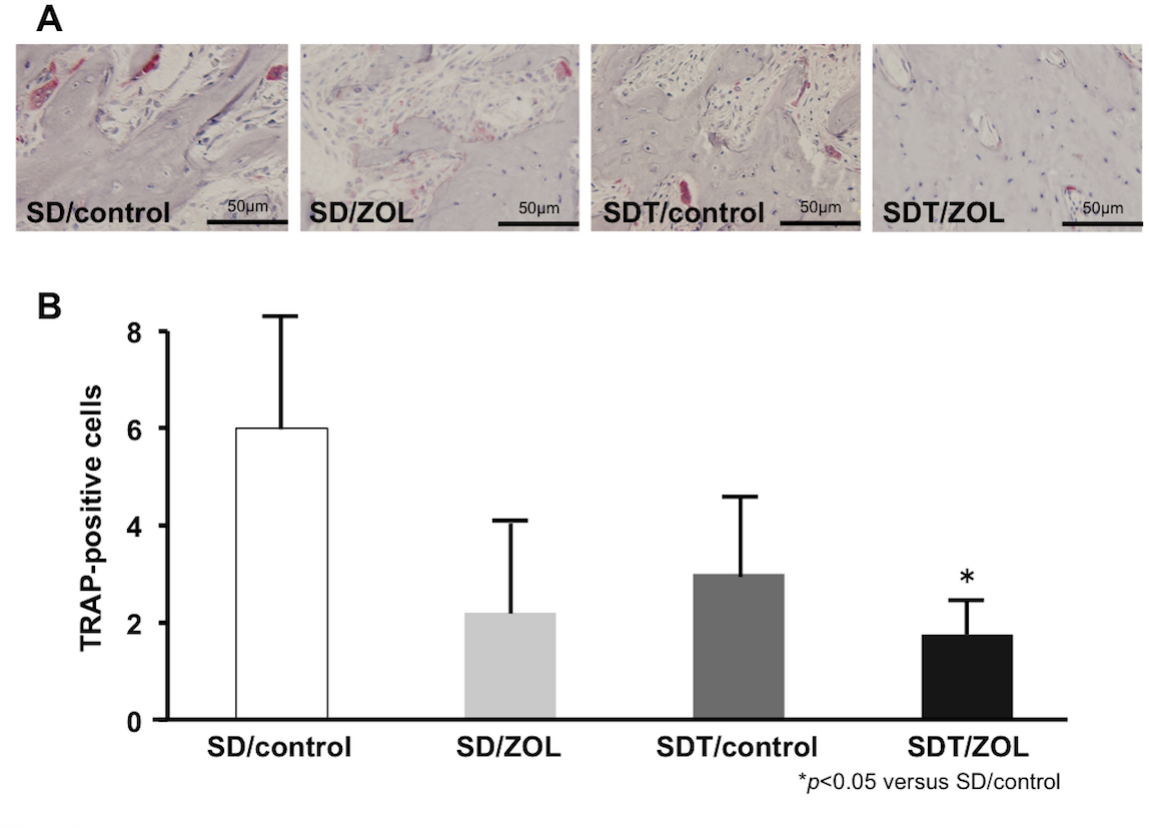
TRAP-stained sections 4 weeks after tooth extraction. **(A)** TRAP-positive osteoclasts on the bone surface in the upper alveolar bone. Original magnification, 200×. **(B)** The number of multinuclear TRAP-positive cells was counted in four non-overlapping fields of alveolar bone at a magnification of 200×.

TRAP-positive osteoclasts were present on the bone surface in the upper alveolar bone in the SDT/control group at 4 weeks after extraction. The number of TRAP-positive osteoclasts was lower in the SDT/ZOL group than in the SD/control group; however, the difference between the SDT/control group and the SDT/ZOL group did not reach statistical significance (Fig 6).

## Discussion

BPs bind avidly to bone mineral, but have no substantial affinity for other tissues. Approximately 40–60% of the administered dose of BPs is distributed to bone, while the remainder is excreted unchanged in the urine. The systemic absorption of oral BPs is low (0.6–1.5% of the administered dose) [17]. The incidence of BRONJ is highest in patients with underlying malignancies who receive high doses of IV BPs (e.g., ZOL, 4 mg IV, every 3–4 weeks) to decrease the risk of skeletal complications of malignancy, and BRONJ may develop in 1–10% of such patients [18]. ZOL has a strong affinity for bone mineral and exerts antiresorptive activity by targeting osteoclasts [19]. Long-term BP treatment seems to be an important risk factor for BRONJ [20–22]. The protocol for ZOL used in this study was IV injection at intervals of every 2 weeks for 5.5–7 months, which was considered long-term treatment. Oral surgical procedures increase the incidence of BRONJ [8,23–26]. Kyrgidis et al. [26] reported a case-control study in which tooth extraction during BP treatment significantly increased the risk (adjusted odds ratio, 16.4) of BRONJ. Tooth extraction is the strongest risk factor for the development of BRONJ in patients receiving BPs. Therefore, tooth extraction is commonly used to induce osteonecrosis in rat models.

Why osteonecrosis only develops in the jaws remains unknown. Animal models may help define the pathophysiology of BRONJ and establish preventative and management strategies. It is difficult to conclude that the use of a BP drug combined with the trauma of extraction constitutes a suitable condition for the occurrence of osteonecrosis without any additional related risk factor or comorbidity, as suggested by some previous studies. Diabetes mellitus [27], corticosteroid therapy, chemotherapy [28–30], immunosuppressive therapy [28], endodontic lesions, periodontal disease, abscesses [23], and poor oral hygiene [10] are considered cofactors for the development of BP-induced osteonecrosis. Therefore, in the presence of treatment with ZOL, one or more of these cofactors might play an essential role in the development of osteonecrosis. Studies to assess this possibility are important because monitoring of these controllable cofactors may contribute to the prevention of osteonecrotic lesions and because such cofactors might constitute a contraindication for BP use [31]. The objective of our study was to establish an animal model of BRONJ by exposure to a combination of risk factors. In this study, diabetes was chosen as one of the risk factors.

Diabetes mellitus is generally associated with microvascular ischemia of bone [32], endothelial cell dysfunction [33], and decreased bone turnover and remodeling [34] as well as induced apoptosis of osteoblasts and osteocytes [35]. *In vivo* and *in vitro* data uniformly support the notion that new bone formation, as well as bone microarchitectural integrity, is altered in the diabetic state, leading to inadequate bone regeneration after injury [36]. In addition, diabetes mellitus is associated with delayed wound healing [37]. BPs may further exacerbate these conditions. In this study, bone exposure in the oral cavity was observed at the tooth extraction site in the SDT/ZOL group (6/6: 100%) at 8 weeks after tooth extraction. Recent meta-analyses have indicated that the relative risk of hip fracture is increased by 1.4–1.7-fold in patients with type 2 diabetes and by 6.3–6.9-fold in those with type 1 diabetes [38,39]. Other skeletal sites have also been found to be at increased risk for fracture in the diabetic population [40,41]. Tao et al. [42] performed micro-CT analysis of femoral trabecular bone in rats with streptozotocin-induced type 1 diabetes and confirmed that diabetes mellitus significantly decreased BV/TV, Tb.Th, and Tb.N and increased Tb.Sp. Fujii et al. [43] showed that bone mineral density and bone strength were significantly lower in SDT rats than in SD rats. Their data supported the idea that type 2 diabetes mellitus is associated with a low turnover of bone. Ohta et al. [44] reported that SDT rats showed increases in total trabecular area and trabecular number and decreased trabecular thickness in cancellous bones of the trabecular tibia, indicating trabecular miniaturization. In the current study, a similar tendency was observed, but there were no differences in bone histomorphometric parameters of the proximal tibia parameters between SD rats and SDT rats. BPs are widely used as the treatment of choice for osteoporosis because they strongly inhibit bone resorption. We showed that treatment with ZOL significantly increased the levels of BV/TV and Tb.N of the tibia and significantly decreased the levels of Tb.Sp in both SD rats and SDT rats. These findings suggested that our experimental protocol generated the expected anticatabolic effect of ZOL in bone. We evaluated the MAR and the number of TRAP-positive osteoclasts. The MAR and the number of TRAP-positive osteoclasts were significantly lower in the SDT/ZOL group than in the SD/control group. TRACP-5 is a serum bone resorption marker reflecting osteoclast activity, has less diurnal variation compared with other bone resorption markers, and is not affected by renal function or diet. Therefore, TRACP-5b might be useful to diagnosis osteoporosis and assess the effectiveness of treatment [45]. In our study, TRACP-5b was significantly lower in the SDT/ZOL group than in SD/control group. These results suggested that both BP treatment and diabetes play important roles in the inhibition of bone turnover in the tooth extraction sockets of rats.

ZOL has been reported to decrease the migration of oral epithelial cells [46], suggesting it disturbs epithelial closure of the tooth extraction socket. After tooth extraction, ZOL deposited in alveolar bone may be released into the tooth socket and affect oral epithelial cells that migrate from the socket edge to cover the extraction wound [46]. Coverage by oral epithelial cells is critical not only for successful wound healing but also for protection of the socket from oral bacterial infection. Moreover, it is likely that the delayed wound healing of the extraction socket causes prolonged exposure of alveolar bone to oral bacteria. Kobayashi et al. [47] showed that ZOL promoted the proliferation of oral bacteria in healthy mice. There were microbial colonies in both the SDT/control and SDT/ZOL groups in our study; nevertheless, our results showed that the development of BRONJ-like lesions after tooth extraction was clearly more common in the SDT/ZOL group than in the SDT/control group. Bacterial infection itself may thus not be critical for the progression of BRONJ.

Inflammation plays an important role in the elimination of contaminating microorganisms and is thus a natural step in the wound-healing process [48]. Recently, increasing *in vitro* and *in vivo* evidence supports the idea that BPs can regulate the immune system by modulating both innate and adaptive immune responses [18,49,50] and by impairing monocyte/macrophage and dendritic cell maturation and function [51,52]. Immunomodulation induced by BP treatment can cause either immunosuppression or a generalized enhanced immune response [53], which may subsequently promote the development of BRONJ.

In the SDT/control group, osteomyelitis was observed at 4 weeks after tooth extraction. At 8 weeks, these osteomyelitic changes had disappeared, the tooth extraction sockets were completely covered by epithelium, and the affected region had begun to heal. In the SDT/ZOL group, however, osteomyelitis persisted until 8 weeks after tooth extraction, clearly indicating incomplete healing. The above findings suggest that osteomyelitis occurred transiently after tooth extraction in the SDT/control group, but was followed by wound healing with epithelial coverage. In contrast, low bone turnover apparently prevented sequestration in the SDT/ZOL group. This was assumed to be the cause of prolonged osteomyelitis.

In conclusion, we established BRONJ-like lesions in a rat model of type 2 diabetes. Investigation of this model showed that BP administration caused delayed wound healing of the mucosal epithelium after tooth extraction, leading to infection in the area that progressed to osteomyelitis. Because there was only a slight inflammatory cell response, it is likely that osteomyelitis was prolonged with low bone turnover in the extraction socket, inhibiting normal bone sequestration. Both BP treatment and diabetes delay mucosal epithelial wound healing, inhibit inflammatory cell responses, and suppress bone turnover. Our results suggest that both of these factors contribute to the onset of BRONJ. Further research on this topic is needed to confirm and extend our findings.
